# Fractionation of Extracts from Black Chokeberry, Cranberry, and Pomegranate to Identify Compounds That Influence Lipid Metabolism

**DOI:** 10.3390/foods11040570

**Published:** 2022-02-16

**Authors:** Sonja Niesen, Celina Göttel, Hanna Becker, Tamara Bakuradze, Peter Winterhalter, Elke Richling

**Affiliations:** 1 Institute of Food Chemistry, Technische Universität Braunschweig, Schleinitzstraße 20, D-38106 Braunschweig, Germany; s.niesen@tu-braunschweig.de (S.N.); p.winterhalter@tu-braunschweig.de (P.W.); 2 Division of Food Chemistry and Toxicology, Department of Chemistry, Technische Universität Kaiserslautern, Erwin-Schrödinger-Straße 52, D-67663 Kaiserslautern, Germany; goettel@chemie.uni-kl.de (C.G.); hbecker@chemie.uni-kl.de (H.B.); bakuradze@chemie.uni-kl.de (T.B.)

**Keywords:** lipid accumulation, lipolysis, phosphodiesterase activity, anthocyanins, copigments, polymers, red fruit juice, HPLC-ESI-MS/MS, HPCCC

## Abstract

Polyphenols show a spectrum of bioactive effects, including an influence on lipid metabolism. In this study, we performed activity-guided fractionations of black chokeberry (aronia), cranberry, and pomegranate extracts to identify the biologically active compounds. The extracts were prepared from fruit juice concentrates with the adsorbent resin Amberlite XAD-7 and were separated into a copigment and an anthocyanin fraction, followed by fractionation into a polymer and monomeric fraction by means of hexane precipitation. For further fractionation of the cranberry and pomegranate copigment fractions, high-performance countercurrent chromatography (HPCCC) was used. The compounds in each fraction were identified by high-performance liquid chromatography/electrospray ionization tandem mass spectrometry (HPLC-ESI-MS/MS), and the quantification was performed by ultra high-performance liquid chromatography-diode array detector (UHPLC-DAD) analyses. Each of the (sub-)fractions was tested in three in vitro assays: phosphodiesterase 3B (PDE) activity, lipid accumulation, and lipolysis in 3T3-L1 cells. The results showed that various fractions and subfractions can inhibit lipid accumulation and PDE activity as well as increase lipolysis, particularly copigments. Overall, our results indicate an influence of polyphenol-rich (sub-)fractions on the lipid metabolism.

## 1. Introduction

It is well known that fruits and vegetables are good sources of vitamins, fiber, and bioactive phytochemicals [1]. Compounds with positive effects on health are present in fruits and can also be found in fruit juices, which are controversially discussed because of their sugar content. An already-published work by Ostberg-Potthoff et al. [2] showed that the consumption of red fruit juices can positively influence sugar metabolism by inhibiting sugar-metabolizing enzymes. In red fruit juices, polyphenols are the most important class of secondary metabolites. Polyphenols consist of many subclasses, such as phenolic acids, flavonoids, stilbenes, and lignans [3]. In the case of chokeberry, cranberry, and pomegranate, the phenolic acids and the flavonoids, especially the anthocyanins, are of particular importance [4].

**Figure 1:**
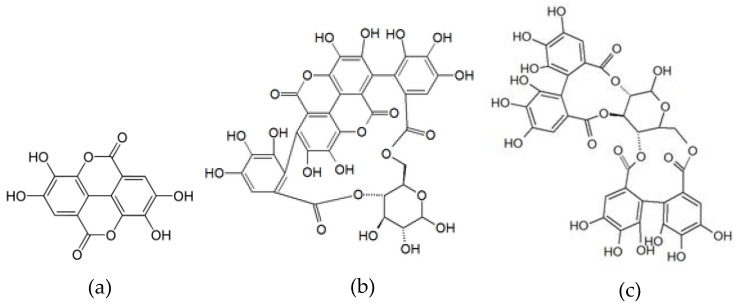
Structure of ellagic acid (**a**), punicalin (**b**), and pedunculagin (**c**).

Pomegranate contains another class of polyphenols, the so called hydrolyzable tannins, which are oligomers of ellagic and gallic acids [5,6]. The monomer ellagic acid and the oligomers punicalin and pedunculagin are shown as examples in Figure 1.

Several studies have shown that polyphenols, especially anthocyanins and copigments, have antioxidant, anti-inflammatory, anti-bacterial, and anti-viral properties and are discussed in the context of influencing glucose and lipid metabolism in vitro as well as in vivo [7,8,9,10,11,12,13,14].

Triglycerides are stored in cytosolic lipid droplets of fat cells (adipocytes) and are used as the main energy reserve. During lipolysis, triglycerides in fat cells are hydrolyzed; as a result, free fatty acids (FFAs) and glycerol are released. Lipolysis rates are regulated through biochemical or hormonal signals in response to changes in the nutritional state. Furthermore, lipolysis can be influenced by polyphenols, such as flavonoids, by inhibiting 3′,5′-cyclic adenosine monophosphate (cAMP)-phosphodiesterase (PDE), such as PDE 3B, which led to increased cAMP levels, as reported by Kuppusamy and Das [15] and Dallas et al. [16]. Cyclic AMP is a second messenger that activates protein kinase A, which leads to increased phosphorylation of hormone-sensitive lipase and perilipin 1. This stimulates the activation of both (hormone-sensitive lipase and perilipin 1) and promotes lipolysis [17,18]. In addition, extracts containing different classes of polyphenols or individual polyphenols have shown that they reduce lipid accumulation via suppressed differentiation and induced lipolysis in 3T3-L1 cells [19,20,21,22].

This paper describes the successful fractionation and identification of compounds from chokeberry, cranberry, and pomegranate extracts, influencing lipid metabolism in vitro. Therefore, we examined the effects of the (sub-)fractions on lipid accumulation, lipolysis, and PDE activity, which represent some parts of human lipid metabolism.

## 2. Materials and Methods

### 2.1. Chemicals and Reagents

All chemicals and reagents used were of analytical grade. Adenosine monophosphate (AMP), and cyclic adenosine monophosphate (cAMP) were purchased from Alexis Biochemicals (Lörrach, Germany); barium hydroxide octahydrate (Ba(OH)_2_ • 8H_2_O), caffeine, Oil Red O, phosphodiesterase (PDE) 3B (recombinant, EC3.1.4.17), resazurin sodium salt, saponine, and butanol (BuOH) were in HPLC grade from Sigma Aldrich (Steinheim Germany); [2,8-^3^H]-3′5′-cyclic adenosine monophosphate ammonium salt, 37 MBq/mL, was from Hartmann Analytic (Braunschweig, Germany). Tris-(hydroxymethyl)-aminomethane (TRIS) was obtained from Carl Roth (Karlsruhe, Germany), and resveratrol was from Cayman Chemical (Ann Arbor, MI, USA). Formaldehyde solution of 37%; magnesium chloride hexahydrate (MgCl_2_ • 6H_2_O); zinc sulphate heptahydrate (ZnSO_4_ • 7H_2_O); and the standards of cyanidin-3-glucoside, quercetin-3-glucoside, chlorogenic acid, and punicalin were ordered from Merck (Darmstadt, Germany). Isopropanol and *tert*-butyl methyl ether (*t*BME) in HPLC grade were purchased from Fisher Scientific GmbH (Schwerte, Germany); dimethyl sulfoxide (DMSO) was from J&K Scientific (Pforzheim, Germany). Acetonitrile (ACN) was purchased in HPLC and LC-MS grade from Honeywell Specialty Chemicals (Seelze, Germany), and methanol (MeOH) was in HPLC grade from VWR Chemicals (Darmstadt Germany). The mouse embryo 3T3-L1 cell line was obtained from Zenbio (Research Triangle Park, NC, USA). Cell culture consumable materials were obtained from Greiner Bio-One (Essen, Germany). The cinnamtannin A2 standard (epicatechin tetramer) was obtained from PhytoLab GmbH & Co. KG (Vestenbergsgreuth, Germany.)

### 2.2. Samples

All extracts were generated using Amberlite^®^ XAD-7 adsorber resin from Sigma Aldrich (Steinheim Germany). The extracts were obtained from authentic juice concentrates from commercial sources: chokeberry with a °Brix of 65.4 and a juice dilution of 18 °Brix, cranberry with 49.8 °Brix and juice dilution of 8.8 °Brix, and pomegranate with 64.5 °Brix and a juice dilution of 15 °Brix.

### 2.3. XAD-7 Extraction

About 2 L of the juice concentrates were applied onto an Amberlite^®^ XAD-7 column. The column was washed with 4 L of water. The retained phenolic compounds were eluted with 2 L of a mixture of methanol/water (19/1 *v*/*v*). Solvents were evaporated under reduced pressure at 40 °C, and the XAD-7 extracts were freeze-dried with a Beta 2-8 LD plus from Christ (Osterode, Germany).

### 2.4. Fractionation by Membrane Chromatography

A Sartobind S IEX 150 mL cellulose membrane (Sartorius, Göttingen, Germany) was used to separate the anthocyanins from the copigments and other phenolic compounds, which will further collectively be called “copigment fraction”. The XAD-7 extracts (approx. 6 g) were dissolved in 1 L of methanol/acetic acid (19/1 *v*/*v*). The membrane was washed with 2 L of NaOH (1 N) and equilibrated with 2 L of HCl (0.01 N) and 1.5 L of methanol/acetic acid (19/1 *v*/*v*). Then, the membrane adsorber was loaded with the dissolved extracts. The copigment fractions were eluted with a further liter of methanol/acetic acid (19/1 *v*/*v*). The retained anthocyanins were eluted with 1 L of a mixture of aqueous NaCl solution (1 N) and methanol (1/1 *v*/*v*). The membrane was washed with 2 L of NaOH (1 N) and equilibrated with a mixture of aqueous NaCl solution (0.1 N) and ethanol (8/2 *v*/*v*). The solvent of the copigment fractions were removed in vacuo, and the residue was freeze-dried. For removal of NaCl from the liquid anthocyanin fraction, a further extraction with Amberlite XAD-7 had to be carried out [23] (see Section 2.3).

### 2.5. Precipitation of the Polymer Polyphenols

Polymer compounds can be precipitated by lipophilic solvents such as hexane. Therefore, the extract must first be solubilized in a hydrophilic solvent such as ethanol. With constant stirring of the dissolved sample, hexane is slowly added dropwise into the solution, so that the polymers precipitate. To some extent, hexane also precipitates the monomers, so that the correct ratio has to be found for each single sample. The precipitate can then be filtered off, the solvents evaporated, and then the precipitate (polymer fraction) can be freeze-dried [24]. The following ratios were used for the precipitation step: chokeberry extract with ethanol/hexane (4/3 *v*/*v*), cranberry extract with ethanol/hexane (1/1 *v*/*v*), and pomegranate extract with ethanol/hexane (2/1 *v*/*v*).

### 2.6. HPLC-ESI-MS/MS Parameters for Identification

The HPLC was from Agilent (Waldbronn, Germany). Pump: Agilent 1100 G1312A binary pump; autosampler: Agilent 1200 G1321B ALS SL; detector: Agilent 1100 G1315B DAD. The software used was Bruker HyStar V. 3.2 (Bremen, Germany), and the mass spectrometer was from Bruker (HCT Ultra Ion Trap with electrospray ionization). The column was an Agilent Zorbax Eclipse Plus C18, 3.5 mm × 150 mm (3.5 µm). The flow rate was 0.4 mL/min at a temperature of 40 °C, with solvent system A = 0.5% formic acid and B = acetonitrile and a gradient as follows: 0 min 1% B, 8 min 1% B, 15 min 5% B, 40 min 20% B, 45 min 30% B, 50 min 95% B, 55 min 1% B, and 60 min 1% B. Compound identification was done by the isocontour plot of the HPLC-ESI-MS/MS analysis from λ 200 to 600 nm and the DAD chromatogram at a wavelength of λ 520 nm for anthocyanins and λ 320 nm and λ 360 nm for the copigments, as well as by mass spectra data.

### 2.7. UHPLC-DAD-Parameters for Quantification

The pump, autosampler, column oven, and DAD detector were from Agilent (Waldbronn, Germany) and belong to the Agilent 1290 Infinity II series; the software was Agilent Open Lab CDS, ChemStation edition, version B.04.03-Sp2. The injection volume was 5 µL, and the measured wavelength was from 200–700 nm. The column was an Agilent Zorbax Eclipse Plus C18, 3.5 mm × 150 mm (1.8 µm).

The conditions for all measurements, with the exception of polymers, were as follows: the flow rate was 0.55 mL/min and the temperature 45 °C. Solvent system A was 0.5% formic acid, and B was 100% acetonitrile with the following gradient: 0 min 1% B, 19 min 5% B, 23 min 30% B, 25 min 95% B, 27 min 95% B, 28 min 1% B, and 30 min 1% B. Quantification was performed with different calibration curves: with cyanidin-3-glucoside for anthocyanins at a wavelength of λ 520 nm, with quercetin-3-glucoside for flavonoids at a wavelength of λ 360 nm, with chlorogenic acid for phenolic acids at a wavelength of λ 320 nm, and with punicalin for hydrolyzable tannins at a wavelength of λ 360 nm.

The conditions for polymers were a flow rate of 0.5 mL/min, a temperature of 35 °C, and solvent A was 2% formic acid, and solvent B 100% acetonitrile with the following gradient: 0 min 5% B, 10 min 5% B, 30 min 20% B, 32 min 40% B, 38 min 60% B, 40 min 70% B, 42 min 80% B, 44 min 100% B, 46 min 100% B, 47 min 5% B, and 55 min 5% B. Quantification was performed with a calibration curve of cinnamtannin A2 at a wavelength of λ 280 nm.

### 2.8. High Performance Counter Current Chromatography (HPCCC)

The HPCCC was a Spectrum 13,020,203 Centrifuge, Dynamic Extractions Ltd. (Berkshire, UK) with a coil volume of 120 mL and an injection volume of 5 mL. The fractionations were performed at a rotation speed of 1600 rpm and in head-to-tail mode, the flow rate was 3 mL/min, and the detection took place at a wavelength of λ 320 nm. Fractions were collected every 2 min.

The solvent system for the cranberry copigment fraction was *t*BME/ACN/H_2_O (2/2/3 *v*/*v*/*v*). The solvent system for the pomegranate copigment fraction was *t*BME/BuOH/ACN/H_2_O (4/2/3/8 *v*/*v*/*v*/*v*) + 0.1% TFA. The HPCCC fractionation of the cranberry copigment fraction extract had to be repeated about seven times because of the small amount of 300 mg that could be injected in order to obtain good fractionation. The separation of the pomegranate copigment fraction was repeated 28 times. The coil fraction was the liquid solution that remained in the HPCCC after completion of the separation.

### 2.9. 3T3-L1 Cell Culture, Differentiation, and Treatment

The 3T3-L1 preadipocytes were grown in preadipocyte medium (Zenbio, Research Triangle Park, NC, USA), and the culture was maintained at 37 °C, 5% CO_2_, and 95% relative humidity.

Due to their potential to differentiate from fibroblasts to adipocytes, 3T3-L1 cells are widely used for studying lipid metabolism. Therefore, cells were grown in 48-well plates (lipid accumulation assay) or 96-well plates (lipolysis assay and alamarBlue assay) at an initial density of 0.9 × 10^4^ cells/cm^2^. Two days post-confluence, the differentiation of the preadipocytes was stimulated by changing the medium to a differentiation medium (Zenbio, Research Triangle Park, NC, USA). After 4 days, the medium was replaced with adipocyte medium (Zenbio, Research Triangle Park, NC, USA). The medium was refreshed at either three- or four-day intervals thereafter until analysis was performed 14 days after differentiation.

For determination of lipid accumulation, the differentiating 3T3-L1 cells were incubated with the fractions or subfractions (*c* = 10–50 µg/mL: chokeberry and cranberry; *c* = 5–25 µg/mL: pomegranate) for 14 days. For the lipolysis experiments, the differentiated adipocytes (14 days post-differentiation) were incubated with the fractions or subfractions at concentrations of 1–4 mg/mL for 3 h. Fractions and subfractions were dissolved in DMSO.

### 2.10. Cytotoxicity (Alamarblue Assay)

Cytotoxicity was determined with the alamarBlue assay, which is used to measure metabolic activity by the reduction of resazurin to resorufin (fluorescent). Thus, cell viability is proportional to cell growth and mitochondrial integrity [25]. After incubation with the fractions or subfractions, either for 14 days (as in the lipid accumulation assay) or for 3 h (as in the lipolysis assay; see Section 2.9), the cells were washed with phosphate buffered saline (PBS) and treated for 1 h with serum-free medium (200 µL) containing 10% resazurin solution. Fluorescence was measured by a microplate reader (Synergy 2, BioTek Instruments GmbH, Bad Friedrichshall, Germany; ex 530 nm, em 590 nm, 37 °C). Saponine (0.1%) was used as a positive control. Results were expressed as relative cell viability as a percentage of that of the solvent control (DMSO).

### 2.11. Determination of Lipid Accumulation by Oil Red O Staining

The lipid content in the differentiated 3T3-L1 adipocytes was determined using the Oil Red O staining method, according to the method published by Kowalska et al. [21] with slight modifications. Briefly, the cells were washed with PBS after incubation, fixed in 250 µL formalin (10%) for 1 h, and washed with 60% isopropanol. The cells were then incubated with 100 µL Oil red O staining solution (0.21% in 60% isopropanol) for 10 min at room temperature, washed with water to remove unbound dye, and left to dry. Fat droplets, stained red, were extracted from cells using 375 µL 100% isopropanol, and the absorbance was measured at a wavelength of 520 nm with a microplate reader (Sirius HT, BioTek Instruments GmbH, Bad Friedrichshall, Germany). Resveratrol (*c* = 11.4 µg/mL and 22.8 µg/mL) was used as a positive control, according to Fischer-Posovsky et al. [26]. Lipid accumulation is presented as the percentage of the solvent control (DMSO; final concentration 0.1%).

### 2.12. Lipolysis Assay

Free fatty acid amounts were used as an indicator of adipocyte lipolysis and determined by using the Lipolysis Assay Kit for 3T3-L1 cells (Zenbio, Research Triangle Park, NC, USA), following the manufacturer´s instructions. Values were corrected by the self-absorption of the test substances. Data are expressed as FFAs released in comparison to that of the solvent control (DMSO; final concentration 1%).

### 2.13. The cAMP-Specific PDE Activity Assay

The inhibitory effect of each fraction and subfraction on cAMP-specific PDE 3B was measured according to the method published by Pöch et al. [27] with slight modifications [28]. Briefly, 100 µL of the sample (five concentrations depending on the inhibition strength) and 20 µL of PDE 3B (200 Units/mL) were incubated for 15 min at 4 °C. After adding 50 µL of cAMP mix (30 mM Tris/HCl pH 7.4, 9 mM MgCl_2_, 3 mM 5′AMP, 3 µM cAMP, and 96.2 kBq/mL [2,8-^3^H]-cAMP), the mixture was incubated for 25 min at 37 °C to allow the reaction. The enzyme reaction was stopped by adding 250 µL of ZnSO_4_ (0.266°M) on ice. [^3^H]-5′-AMP was then precipitated by adding 250 µL of Ba(OH)_2_ (0.266°M). The tubes were then centrifuged at 13,000× *g* for 9 min at 25 °C, 450 µL of the supernatant was mixed with 3.5 mL of a scintillation cocktail, and the resulting radioactivity was measured using a liquid scintillation counter (Wallac 1410, Pharmacia, Uppsala, Sweden). Samples were dissolved in DMSO (final concentration of 1%) or in assay buffer. Caffeine, the nonspecific PDE 3B inhibitor, served as a positive control. The experiments were performed in triplicate, and the half-maximal inhibitory concentration (IC_50_) values were determined after at least three independent experiments.

### 2.14. Statistical Analysis

The results of the in vitro assays were presented as the mean ± standard deviation (SD) of at least three independent experiments. Statistical analyses were conducted using the Analysis Tool Excel of Microsoft 365 Apps for Enterprise (Microsoft Corporation, Redmond, WA, USA) and Origin 2020 (OriginLab, Northampton, MA, USA). The data of samples treated with fractions or subfractions were analyzed for significant differences (*p* < 0.05, *p* < 0.01, and *p* < 0.001) compared to the solvent-treated control by a Student’s *t*-test (one-sided).

## 3. Results

### 3.1. Fractionation of the Extracts of Chokeberry, Cranberry, and Pomegranate by Membrane Chromatography

First, extracts (see Section 2.1) were prepared from concentrates. These extracts contained polyphenolic compounds and were free of carbohydrates, minerals, and other components interfering with the assays used. The amount of dilution depended on the °Brix values, which were determined for each fruit juice by the Official Journal of the European Union [29]. The °Brix values correlated with the sugar content. The concentrates had an extract content and a calculated content for juice dilution, as shown in Table 1.

Chokeberry revealed the highest polyphenolic content, with 4.08 g/100 mL, followed by pomegranate, with 2.31 g/100 mL, and cranberry, with 1.39 g/100 mL.

The next step was the fractionation of the extracts by membrane chromatography into the anthocyanin and copigment fractions (see Section 2.2). In this process, polymeric compounds were almost removed. The contents of anthocyanins and copigments in the extracts are shown in Table 2.

### 3.2. Qualification and Quantification of Polyphenolic Compounds in Anthocyanin and Copigment Fractions

#### 3.2.1. Chokeberry

**Figure 2:**
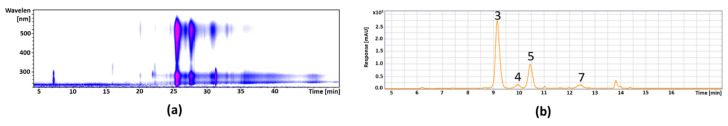
Isocontour plot of the HPLC-ESI-MS/MS analysis from λ 200 to 600 nm (**a**) and a DAD chromatogram of UHPLC separation at a wavelength of λ 520 nm (**b**) from the chokeberry anthocyanin fraction. The numbers are defined in the Table of the HPLC-ESI-MS/MS data (Table 3).

**Figure 3:**
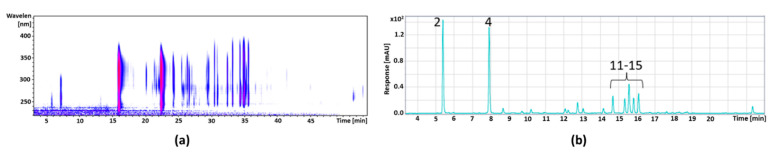
Isocontour plot of the HPLC-ESI-MS/MS analysis from λ 200 to 450 nm (**a**) and a DAD chromatogram of UHPLC separation at a wavelength of λ 360 nm (**b**) from the chokeberry copigment fraction. The numbers are defined in the Table of the HPLC-ESI-MS/MS data (Table 4).

The identification of the polyphenols in the extracts was carried out using HPLC-ESI-MS/MS (see Section 2.3) and the quantification using UHPLC-DAD (see Section 2.4). Figure 2 shows the isocontour plot and the DAD chromatogram of the chokeberry anthocyanin fraction. Peak identification and quantification are provided in Table 3. The isocontour plot and the DAD chromatogram of the chokeberry copigment fraction are presented in Figure 3, and the peak identification and quantification are in Table 4. Compounds were identified by mass spectral data, comparisons with authentic references (marked with *), and literature data [30,31].

#### 3.2.2. Cranberry

**Figure 4:**
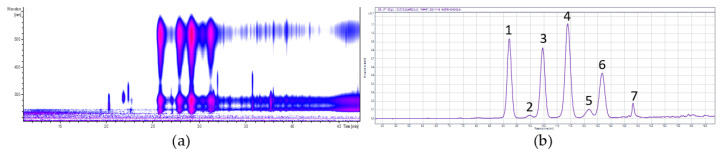
Isocontour plot of the HPLC-ESI-MS/MS analysis from λ 200 to 600 nm (**a**) and a chromatogram of UHPLC-DAD separation and detection at a wavelength of λ 520 nm (**b**) of the cranberry anthocyanin fraction.

**Figure 5:**
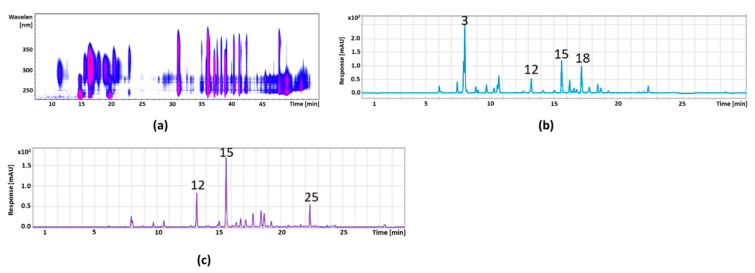
Isocontour plot of the HPLC-ESI-MS/MS analysis from λ 200 to 450 nm (**a**) and a DAD chromatogram of the UHPLC separation at a wavelength of λ 320 nm (**b**) and λ 360 nm (**c**) from the cranberry copigment fraction.

Figure 4 shows the isocontour plot and the DAD chromatogram of the cranberry anthocyanin fraction, whereas the peak identification and quantification are provided in Table 5. In Figure 5, the isocontour plot and the DAD chromatogram of the cranberry copigment fraction are shown. In Table 6, a summary of the peak identification and quantification is provided. The compounds were identified by mass spectral data, comparisons with authentic references (marked with *), and literature data [32].

#### 3.2.3. Pomegranate

**Figure 6:**
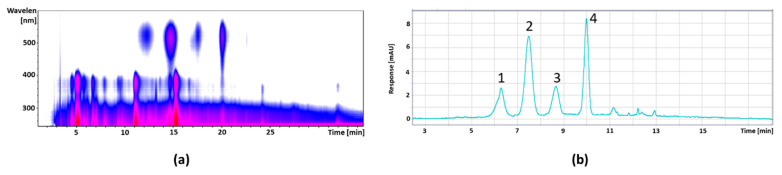
Isocontour plot of the HPLC-ESI-MS/MS analysis from λ 200 to 600 nm (**a**) and a DAD chromatogram of the UHPLC separation at a wavelength of λ 520 nm (**b**) from the pomegranate anthocyanin fraction.

**Figure 7:**
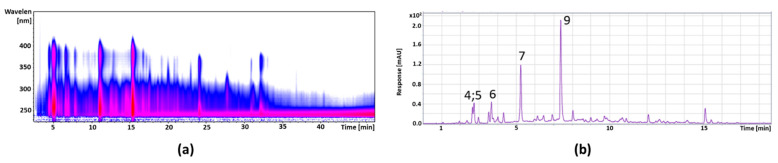
Isocontour plot of the HPLC-ESI-MS/MS analysis from λ 200 to 500 nm (**a**) and a DAD chromatogram of UHPLC separation at a wavelength of λ 360 nm (**b**) from the pomegranate copigment fraction.

Figure 6 shows the isocontour plot and the DAD chromatogram of the pomegranate anthocyanin fraction; the peak identification and quantification are given in Table 7. Next follows the isocontour plot and the DAD chromatogram of the pomegranate copigment fraction in Figure 7 and the peak identification and quantification in Table 8. The compounds were identified by mass spectral data, comparisons with authentic reference (marked with *), and literature data [33,34].

### 3.3. Polymer Content of the Extracts of Chokeberry, Cranberry, and Pomegranate

**Figure 8:**
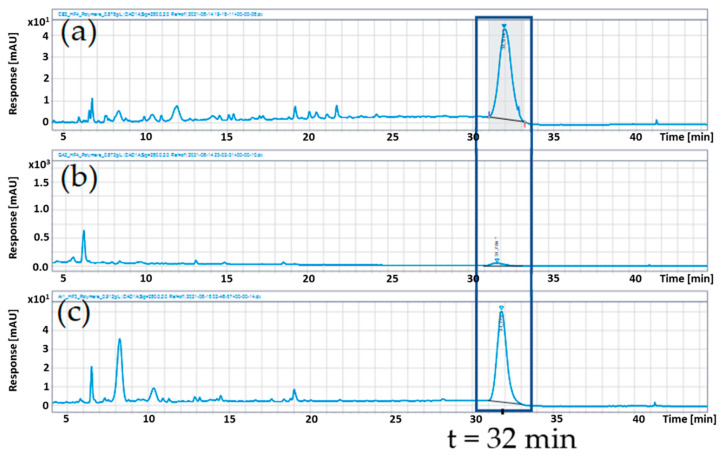
UHPLC-DAD chromatograms at λ 280 nm of the polymer fractions of cranberry (**a**), pomegranate (**b**), and chokeberry (**c**) extracts, with the polymer peak at a retention time of 32 min.

The polymer content was determined by hexane precipitation. Figure 8 shows the UHPLC-chromatograms after precipitation. The polymers were quantified with a calibration curve as cinnamtannin A2 equivalents, which is an epicatechin tetramer. Table 9 lists the polymer contents in the precipitates as well as in the original extracts.

The hexane precipitates only enriched the polymers, because there was always a balance between monomers, oligomers and polymers, but compared to the extracts, an accumulation took place after the precipitation. The cranberry extract showed the highest polymer content after precipitation with 41.2 g/100 g, followed by chokeberry extract with 37.8 g/100 g. The pomegranate extract revealed a very low polymer content of 5.3 g/100 g. These values are to be regarded as cinnamtannin A2 equivalents, so that the actual polymer content could be higher.

### 3.4. Effects of the Three Fractions (Anthocyanins, Copigments, and Polymers) on Cytotoxicity and Lipid Metabolism In Vitro

#### 3.4.1. Cytotoxicity

The effects on cell viability of 3T3-L1 cells after treatment with the anthocyanin, copigment, and polymer fractions of chokeberry, cranberry, and pomegranate extracts were determined by the alamarBlue assay.

To evaluate non-cytotoxic concentration ranges for the lipid accumulation assay, the cells were incubated for 14 days (as in the lipid accumulation assay) with the chokeberry, cranberry (each *c* = 10–100 µg/mL), as well as pomegranate fractions (*c* = 5–100 µg/mL). All tested fractions (anthocyanins, copigments, and polymers) reduced cell viabilities in a concentration-dependent manner. A cell viability of around 80% was observed for all fractions of chokeberry and cranberry extracts at concentrations of 10–25 µg/mL and pomegranate extract at concentrations of 5–10 µg/mL (data not shown). In general, the pomegranate fractions showed stronger effects on cell viability than the fractions produced from chokeberry or cranberry extracts, and the cell viability decreased in the order of chokeberry ≈ cranberry > pomegranate. Based on this screening, the following concentration ranges for the lipid accumulation assay were selected: the fractions of chokeberry and cranberry extract were tested in concentrations of 10–50 µg/mL and the fractions of pomegranate extract in concentrations of 5–25 µg/mL.

Since, for the lipolysis assay, the cells were incubated for 3 h, differentiated adipocytes (14 days post-differentiation) were treated with fractions at concentrations of 1–4 mg/mL. At lower concentrations (*c* = 1–2 mg/mL), a cell viability of 80–100% was observed, while at higher concentrations (*c* = 3–4 mg/mL; data not shown), a decrease of up to 65% was shown.

#### 3.4.2. Lipid Accumulation

Adipocyte differentiation is a process of morphological changes of preadipocytes to become differentiated adipocytes: rounded-shape cells with intracellular lipid accumulation. Therefore, the total content of lipid accumulation can indicate adipocyte differentiation and can be detected by using the Oil Red O staining technique [35].

**Figure 9:**
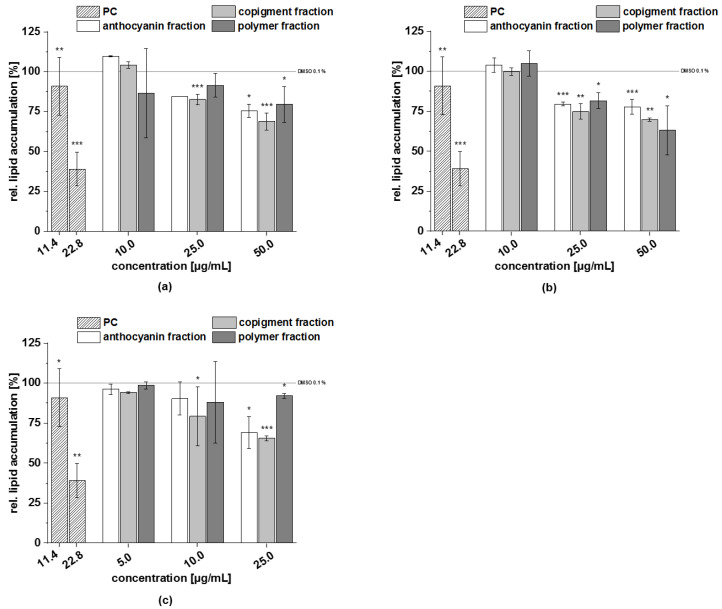
Effects of the three fractions (anthocyanin, copigment, and polymer) of chokeberry (**a**), cranberry (**b**), and pomegranate (**c**) extracts on lipid accumulation in 3T3-L1 cells by Oil Red O staining. Fractions were added to the 3T3-L1 cell cultures at the stage of the differentiation process for 14 days at concentrations of 10–50 µg/mL or 5–25 µg/mL. The positive control (PC) was resveratrol. Data are the mean values ± standard deviation (*n* = 3–11). The significance of differences between sample and solvent control (100%) was assessed using a Student’s *t*-test. * *p* < 0.05; ** *p* < 0.01; *** *p* < 0.001.

Resveratrol can inhibit adipocyte differentiation of 3T3-L1 cells [36] and was used as a positive control (PC) in this assay. The test results of the fractions (anthocyanins, copigments, and polymers) are presented in Figure 9. All fractions of the chokeberry extract did not significantly modulate lipid accumulation at a concentration of 10 µg/mL; however, the relative lipid accumulation at higher concentrations was reduced to approximately 85% (*c* = 25 µg/mL) and 75% (*c* = 50 µg/mL), respectively, compared to the solvent control (100% lipid accumulation). Dose-dependent effects were detected with the fractions of the cranberry extract. The relative lipid content was decreased by up to 63% following treatment with the cranberry polymer fraction at a concentration of 50 μg/mL (Figure 9b). The pomegranate anthocyanin and copigment fractions showed similar effects on the relative lipid accumulation: both fractions (*c* = 25 µg/mL) reduced lipid content to approximately 69% and 65% compared to the control, while the polymer fraction showed just a slight effect.

#### 3.4.3. Lipolysis

**Figure 10:**
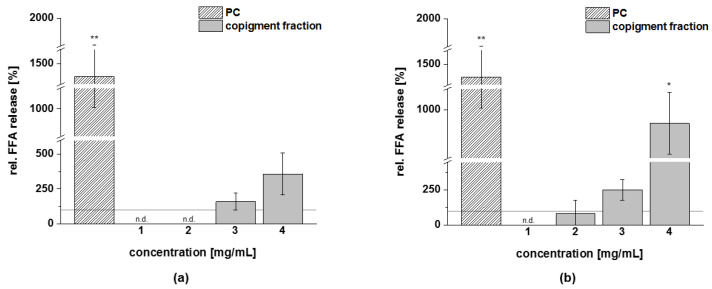
The effects of the copigment fractions of chokeberry (**a**) and cranberry (**b**) extracts on 3T3-L1 adipocyte lipolysis (FFA kit). Differentiated adipocytes were treated for three hours. The conditioned medium was removed from each well and assayed for the relative (rel.) free fatty acid (FFA) content (in percentage of solvent control). The positive control (PC) was isoproterenol (211 ng/mL). Values are expressed as means ± standard deviation (*n* = 1–5). The significance of differences between the sample and solvent control (100%) was assessed using a Student’s *t*-test. * *p* < 0.05; ** *p* < 0.01. n.d. = non-determinable.

Adipocytes were incubated with the fractions of chokeberry, cranberry, and pomegranate extracts to investigate whether they might reduce lipid content by increasing FFA release, which can be used as an indicator of adipocyte lipolysis. Isoproterenol was used as a positive control (PC) in this assay, since it stimulated lipolysis via beta adrenergic receptor activation and cAMP-dependent signaling [37]. The results (Figure 10) confirmed that isoproterenol stimulates lipolysis significantly. For the extract fractions under study, at a concentration of 3 mg/mL, the chokeberry copigment fraction increased lipolysis in a range of 1.6-fold greater than the solvent control, and at a concentration of 4 mg/mL, the FFA release was 3.6-fold greater. The cranberry copigment fraction induced FFA release in a dose-dependent manner of up to 9-fold greater than the solvent control. It could not be excluded that the effects observed in 3T3-L1 cells might have been amplified by reduced cell viability at the highest tested concentrations of the fractions. All other fractions (anthocyanin and polymer) of the extracts and the pomegranate copigment fraction did not significantly increase FFA release in comparison to the control (data not shown).

#### 3.4.4. PDE 3B Activity

The obtained fractions of chokeberry, cranberry, and pomegranate extracts were tested for their influence on cAMP-specific PDE 3B activity in vitro. Data are presented as the relative PDE activity in a percentage of the solvent control. Caffeine (*c* = 235 µg/mL) was used as the positive control (PC) in this assay, since it is a well-known nonspecific PDE 3 inhibitor.

**Figure 11:**
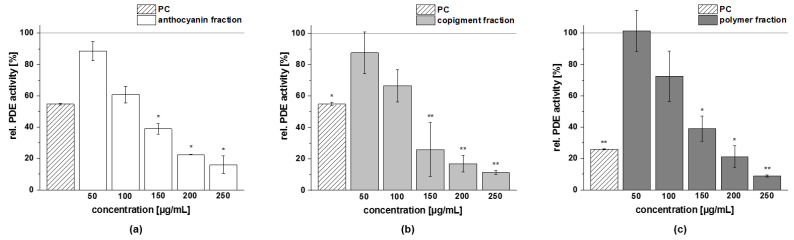
Results of phosphodiesterase 3B (PDE 3B) activity inhibition after incubation with anthocyanin (**a**), copigment (**b**), and polymer (**c**) fractions of the chokeberry extract. The positive control (PC) was caffeine (235 µg/mL). Data are expressed as relative (rel.) PDE activity (in a percentage of the solvent control) as the mean ± standard deviation of three independent experiments. The significance of differences between the sample and solvent control (100%) was assessed using a Student’s *t*-test. * *p* < 0.05; ** *p* < 0.01.

All the tested fractions showed a dose-dependent inhibition of the enzyme PDE 3B in vitro (Figure 11). The PDE 3B activities of the three fractions (anthocyanin, copigment, and polymer) of the chokeberry extract (*c* = 50–250 µg/mL) were in the same concentration range. Relative PDE 3B activities of 60–80% were shown by the chokeberry fractions at a concentration of 100 µg/mL. The highest tested concentration of these fractions (*c* = 250 µg/mL) decreased PDE activity to 16 ± 6%, 11 ± 1%, and 9 ± 1%, respectively, compared to the solvent control (Figure 11).

**Figure 12:**
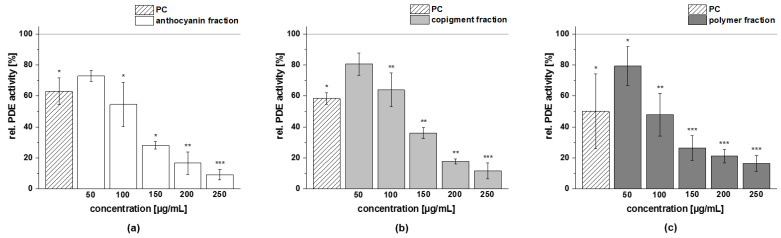
Results of phosphodiesterase 3B (PDE 3B) activity inhibition after incubation with anthocyanin (**a**), copigment (**b**), and polymer (**c**) fractions of the cranberry extract. The positive control (PC) was caffeine (235 µg/mL). Data are expressed as relative (rel.) PDE activity (in a percentage of the solvent control) as the mean ± standard deviation of three or four independent experiments. The significance of differences between the sample and solvent control (100%) was assessed using a Student’s *t*-test. * *p* < 0.05; ** *p* < 0.01; *** *p* < 0.001.

A similar reduced activity of the PDE 3B enzyme activity was observed for the three fractions of the cranberry extract compared to the solvent control (Figure 12). At the 100 µg/mL concentration, the inhibitory activity of the anthocyanin, copigment, and polymer fractions were comparable to the PC caffeine (around 50% PDE activity).

**Figure 13:**
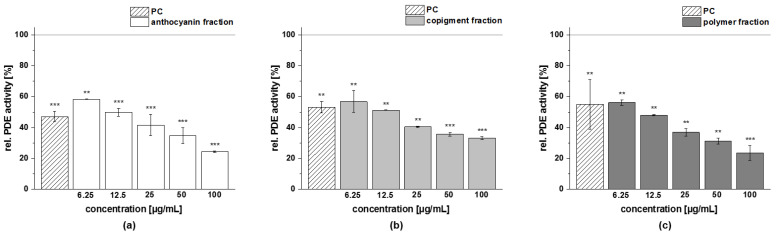
Results of phosphodiesterase 3B (PDE 3B) activity inhibition after incubation with the anthocyanin (**a**), copigment (**b**), and polymer (**c**) fractions of the pomegranate extract. The positive control (PC) was caffeine (235 µg/mL). Data are expressed as relative (rel.) PDE activity (in a percentage of the solvent control) as the mean ± standard deviation of three independent experiments. The significance of differences between sample and solvent control (100%) was assessed using a Student’s *t*-test. ** *p* < 0.01; *** *p* < 0.001.

The three fractions of the pomegranate extract showed the strongest impact on PDE 3B enzyme activity. For the anthocyanin fraction, a significantly diminished PDE activity at the lowest tested concentration (*c* = 6.25 µg/mL) was observed, and the impact on PDE activity was comparable to those seen with the PC caffeine (around 50% PDE activity; Figure 13a). A similar tendency as for the anthocyanin fraction was observed for the pomegranate copigment and polymer fraction. The copigment fraction reduced PDE 3B activity in a dose-dependent manner from 57 ± 7% at the lowest tested concentration (*c* = 6.25 µg/mL) to 33 ± 1% (*c* = 100 µg/mL).

Based on the results of the PDE 3B activity assays, a half-maximal inhibitory concentration (IC_50_) was calculated for each of the fractions investigated (Table 10). The fractions (anthocyanin, copigment, and polymer) of the pomegranate extract were the most active of the tested samples, exhibiting IC_50_ values between 10 µg/mL and 13 µg/mL. The fractions of the chokeberry and cranberry extracts demonstrated a comparable inhibitory potential and revealed nearly similar effects against PDE 3B (IC_50_ = 94–126 µg/mL).

### 3.5. Subfractionation of the Cranberry and Pomegranate Copigment Fraction by High Perfomance Counter Current Chromatography (HPCCC)

Since the copigment fractions of the cranberry and pomegranate extracts showed the highest influence on lipid metabolism, both fractions were further fractionated by high performance counter current chromatography.

#### 3.5.1. Fractionation of Cranberry Copigments by HPCCC

**Figure 14:**
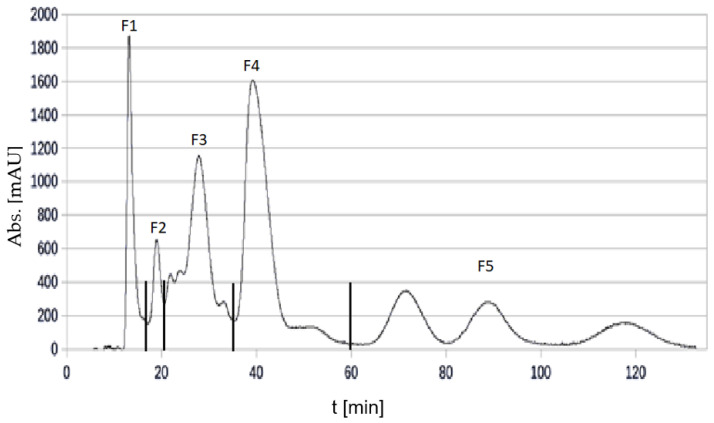
UV-chromatogram at λ 320 nm of the fractionation of the cranberry copigment fraction by HPCCC.

The separation of the cranberry copigment fraction by HPCCC had to be repeated about seven times because of the small amount (300 mg) that could be injected in order to obtain a good fractionation. The total amount that had been separated was about 2.2 g. One of the HPCCC chromatograms is presented in Figure 14, and the fractionation is shown in the chromatogram.

Five subfractions (F1–F5), and the so-called coil fraction, could be collected. The coil fraction was the liquid solution that remained in the HPCCC after completion of the separation and could be collected after separation. Due to the low amount, the coil fraction of the cranberry copigment separation was not further examined. The amount and yield of each fraction are given in Table 11.

#### 3.5.2. Fractionation of the Pomegranate Copigments by HPCCC

**Figure 15:**
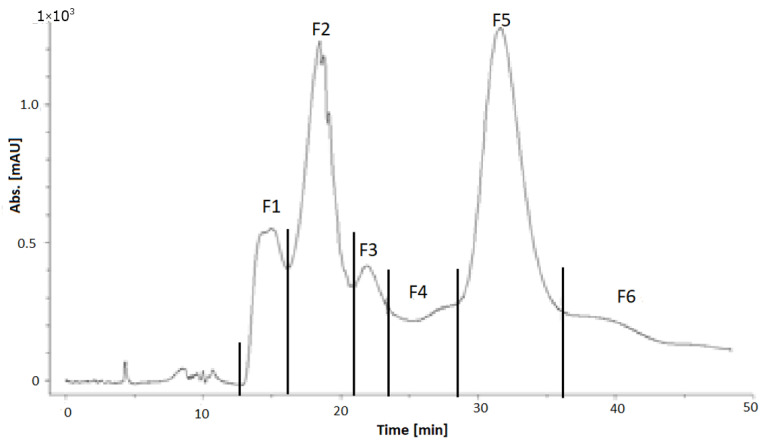
UV-chromatogram at λ 320 nm of the fractionation of the pomegranate copigment fraction by HPCCC.

The separation of the pomegranate copigment fraction by HPCCC had to be repeated 28 times because, for a successful fractionation, only 200–300 mg could be injected. The total injected amount was about 6.1 g. One of the HPCCC chromatograms is presented as an example in Figure 15, and the fractionation is shown in the chromatogram. Six subfractions (F1–F6) and the coil fraction (F7) could be collected. The amount and yield of each fraction are given in Table 12.

#### 3.5.3. Characterization of the HPCCC Subfractions

The characterization of the HPCCC subfractions was performed by HPLC-DAD-MS/MS analyses. The identified compounds are listed in Table 13.

#### 3.5.4. Effects of the Subfractions of the Cranberry Copigment Fraction on Cytotoxicity and Lipid Metabolism In Vitro

First, we determined the effects on cell viability of 3T3-L1 cells after treatment with the subfractions of the cranberry copigment fraction by the alamarBlue assay. After treating 3T3-L1 cells for 14 days with subfraction F1, a dose-dependent reduction of viability in comparison to the solvent control was observed; however, when the cells were treated with concentrations of 10–50 µg/mL of the subfractions F2–F5, no significant cytotoxicity was observed (cell viability ≥80%; data not shown). Based on this screening, a concentration range of 10–50 µg/mL was used for the determination of lipid accumulation.

**Figure 16:**
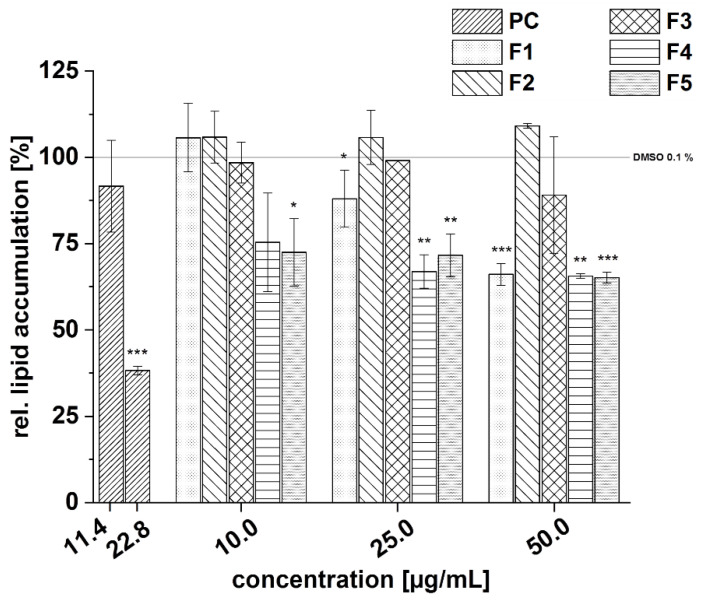
Effects of the five subfractions (F1–F5) of the cranberry copigment fraction on lipid accumulation in 3T3-L1 cells by Oil Red O staining. Subfractions were added to the 3T3-L1 cell cultures at the stage of the differentiation process for 14 days at concentrations of 10–50 µg/mL. The positive control (PC) was resveratrol. Data are the mean values ± standard deviation (*n* = 3). The significance of differences between the sample and solvent control (100%) was assessed using a Student’s *t*-test. * *p* < 0.05; ** *p* < 0.01; *** *p* < 0.001.

The positive control resveratrol reduced lipid accumulation significantly at a concentration of 22.8 µg/mL, compared to the solvent control. Subfractions F4 and F5 were the most active subfractions in reducing lipid accumulation (Figure 16). In the case of subfraction F1, a dose-dependent inhibition of lipid accumulation was observed. No inhibition of lipid accumulation was shown for the subfractions F2 and F3.

The five subfractions (F1–F5) of the cranberry copigment fraction did not stimulate lipolysis of differentiated adipocytes (3T3-L1 cells) by increasing FFA release (data not shown).

**Figure 17:**
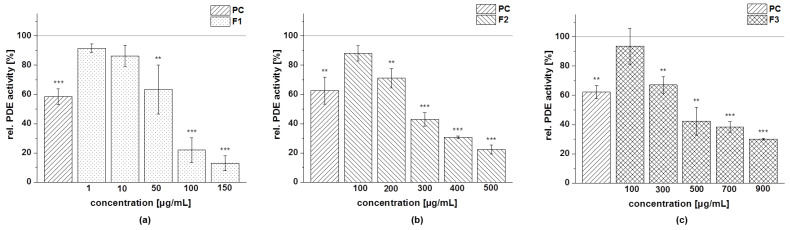

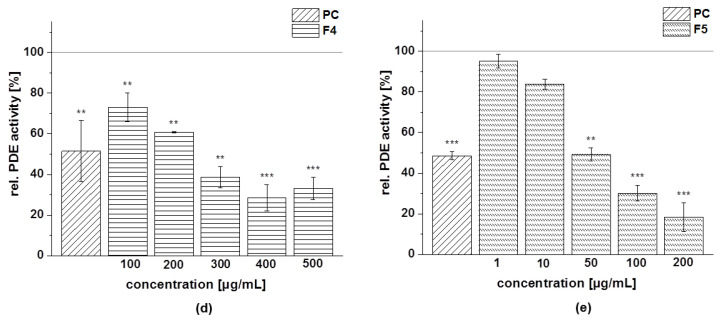
Results of phosphodiesterase 3B (PDE 3B) activity inhibition after incubation with subfractions of the cranberry copigment fraction; F1 (**a**), F2 (**b**), F3 (**c**), F4 (**d**), and F5 (**e**). The positive control (PC) was caffeine (235 µg/mL). Data are expressed as relative (rel.) PDE activity (in a percentage of the solvent control) as the mean ± standard deviation of three or four independent experiments. The significance of differences between the sample and solvent control (100%) was assessed using a Student’s *t*-test. ** *p* < 0.01; *** *p* < 0.001.

The results of the testing of the five subfractions of the cranberry copigment fraction on PDE 3B activity are presented in Figure 17. All compounds showed dose-dependent inhibition of the enzyme PDE 3B in comparison to the solvent control (100% PDE activity) but to varying degrees. The subfractions F1 and F5 were found to be more potent PDE inhibitors than F2 and F4, followed by subfraction F3. Nevertheless, PDE activity was also diminished in a concentration-dependent manner after treatment with F3, and a minimum activity of around 30% was observed at the highest concentration tested (900 µg/mL; Figure 17c).

We calculated the IC_50_ values of the subfractions of the cranberry copigment fraction that we evaluated based on the results of the PDE 3B activity assay (Table 14). The subfractions F1 and F5 were the most potent inhibitors, with IC_50_ values of 45 μg/mL and 37 µg/mL, followed by F2 (IC_50_ = 266 μg/mL) and F4 (IC_50_ = 229 μg/mL). A weaker effect on PDE inhibition was shown by F3, and an IC_50_ value of 471 μg/mL was calculated.

#### 3.5.5. Effects of the Subfractions of the Pomegranate Copigment Fraction on Cytotoxicity and Lipid Metabolism In Vitro

The seven subfractions (F1–F7) of the pomegranate copigment fraction were tested in the different in vitro assays, too. Since the copigment fraction of pomegranate extract did not induce FFA release in 3T3-L1 cells, we did not test the subfractions in the lipolysis assay.

There was minimal cytotoxicity of all seven subfractions treated with low concentrations (*c* = 5–10 µg/mL), with relative viabilities ≥78%. Treatment with concentrations of 25 µg/mL significantly decreased the viability of 3T3-L1 cells compared with the solvent control (data not shown). For the determination of lipid accumulation, the same concentration ranges of 5–25 µg/mL as for the fractions of the pomegranate extract, were used for the subfractions.

**Figure 18:**
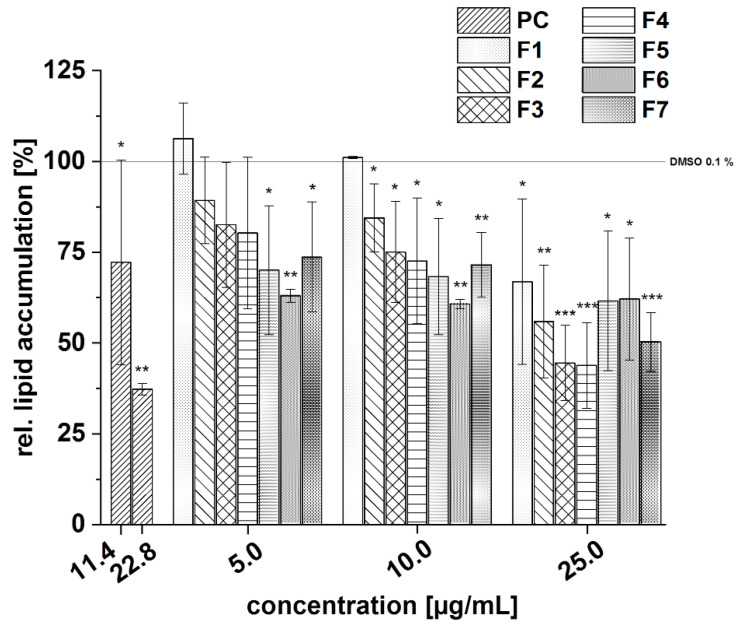
Effects of the seven subfractions (F1–F7) of the pomegranate copigment fraction on lipid accumulation in the 3T3-L1 cells by Oil Red O staining. The subfractions were added to the 3T3-L1 cell cultures at the stage of the differentiation process for 14 days at concentrations of 5–25 µg/mL. The positive control (PC) was resveratrol. Data are the mean values ± standard deviation (*n* = 3). The significance of differences between the sample and solvent control (100%) was assessed using a Student’s *t*-test. * *p* < 0.05; ** *p* < 0.01; *** *p* < 0.001.

All subfractions tended to reduced lipid accumulation in 3T3-L1 cells in the following order of potency: F3 ≈ F4 > F2 ≈ F5 ≈ F6 ≈ F7 > F1 (Figure 18), respectively. The subfractions F3 and F4 showed the strongest inhibitory potential and reduced lipid accumulation to approximately 70% at a concentration of 10 µg/mL, compared to the solvent control.

**Figure 19:**
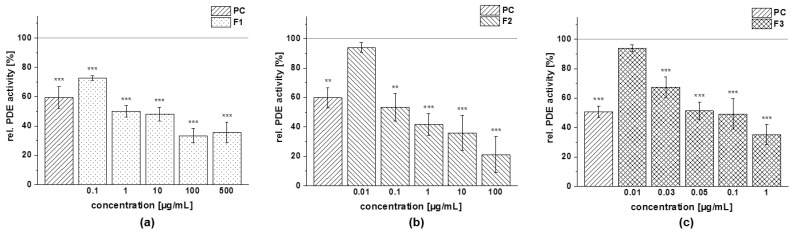

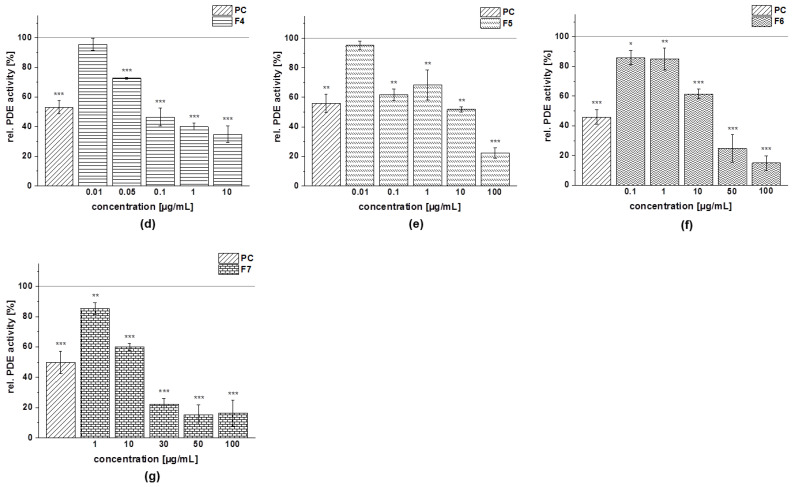
Results of phosphodiesterase 3B (PDE 3B) activity inhibition after incubation with subfractions of the pomegranate copigment fraction; F1 (**a**), F2 (**b**), F3 (**c**), F4 (**d**), F5 (**e**), F6 (**f**), and F7 (**g**). The positive control (PC) was caffeine (235 µg/mL). Data are expressed as relative (rel.) PDE activity (in a percentage of the solvent control) as the mean ± standard deviation of three to five independent experiments. The significance of the differences between the sample and solvent control (100%) was assessed using a Student’s *t*-test. * *p* < 0.05; ** *p* < 0.01; *** *p* < 0.001.

In addition, the inhibitory effects of the subfractions of the pomegranate copigment fraction on PDE 3B activity were investigated in vitro. All tested subfractions (F1–F7) were able to reduce PDE activity (Figure 19). Subfractions F2, F3, and F4 showed the strongest inhibitory effect, whereas a weaker impact on PDE 3B activity was observed by the subfractions F1, F5, F6, and F7. Nevertheless, PDE 3B enzyme activity was also diminished, and a minimum activity of around 15% was observed for the subfraction F7 at the highest concentration tested (*c* = 100 µg/mL; Figure 19g).

The IC_50_ values of the subfractions of the pomegranate copigment fraction are listed in Table 15 and showed that IC_50_ values less than 10 μg/mL were calculated. The less active ones were the subfractions F6 and F7, with IC_50_ values of 8.5 µg/mL and 9.0 µg/mL. The subfractions F1 (IC_50_ = 4.8 µg/mL) and F5 (IC_50_ = 3.8 µg/mL) showed a comparable inhibition level. All other fractions revealed nearly the same inhibitory potential against PDE, i.e., subfraction F2 (IC_50_ values of 0.5 µg/mL), F3 (0.2 µg/mL), and F4 (0.5 µg/mL), respectively.

## 4. Discussion

Within the last years, it has been reported that polyphenols can influence the lipid metabolism in vitro and in vivo [8,16,38,39]. In our previous work, extracts from red fruit juices or concentrates like cranberry, red grape, or blueberry have proven to be potent inhibitors of the enzyme PDE 3B [28]. To identify fruit juice constituents which influence lipid metabolism, a fractionation of the three most potent extracts from chokeberry, cranberry, and pomegranate was performed, and the effects of the anthocyanin, copigment, and polymer fractions in three different in vitro assays (lipid accumulation, lipolysis, and PDE 3B activity) were investigated.

First of all, XAD-7 extracts of commercially available concentrates from chokeberry, cranberry, and pomegranate were generated. These extracts were separated into an anthocyanin and a copigment fraction by membrane chromatography [23]. It was found that the copigment fractions made up the greater part in the extracts of cranberry, with 72.2%, and of pomegranate, with 79.8%. In the chokeberry extract, the anthocyanin fraction predominated, with 47.9%, in comparison to the copigment fraction, with 36.9%. The chokeberry anthocyanin fraction only contained cyanidin derivatives (cyanidin-3-galactoside and cyanidin-3-arabinoside). In the copigment fraction, neochlorogenic acid, chlorogenic acid, and different quercetin derivatives dominated. In the cranberry anthocyanin fraction, cyanidin-, peonidin-pentosides and -hexosides were identified. Peonidin-3-galactoside showed the highest concentration, followed by cyanidin-3-galactoside and cyanidin-3-arabinoside. In the copigment fraction, several phenolic acids, such as feruloylquinic acid, chlorogenic acids, and coumaroylic acids, and several flavonols, such as myricetin, laricetin, quercetin, syringetin, and isorhamnetin derivatives, were present. The compounds in the copigment fraction with the highest concentration were quercetin-hexoside and coumaroyl-hexoside. The pomegranate anthocyanin fraction contained cyanidin and delphinidin mono- and diglucosides. The major compounds were cyanidin-3,5-diglucoside and delphinidin-3-glucoside. The variety of the copigments was much larger and consisted mainly of hydrolyzable tannins of epigallic and gallic acid glycosides, like pedunculagin and punicalagin. These results are in-line with previously published work [30,31,33,34,40]. The polymers were also separated from the extracts. A complete separation could not be obtained, only an enrichment of the polymers.

We could demonstrate that all the tested fractions influenced lipid metabolism by decreasing lipid accumulation and inhibition of PDE 3B activity, whereas only the copigment fractions of the chokeberry and cranberry extracts tended to increase FFA release by lipolysis. Based on the effects of the in vitro assay, the fractions ranked as follows: pomegranate anthocyanin fraction ≈ pomegranate copigment fraction > pomegranate polymer fraction > cranberry polymer fraction > cranberry copigment fraction ≈ cranberry anthocyanin fraction ≈ chokeberry anthocyanin fraction ≈ chokeberry copigment fraction > chokeberry polymer fraction. However, when evaluating the relevance of these results, it could not be excluded that the effects observed in 3T3-L1 cells might have been amplified by reduced cell viability at the highest tested concentrations of the fractions.

Some in vitro studies have also examined the effects of polyphenol-rich extracts on lipid metabolism, as well as one of our previous studies [20,28,41]. Kowalska et al. revealed that a berry fruit extract obtained from chokeberry, raspberry, bilberry, and cranberry fruits (*c* = 25–100 µg/mL) significantly decreased lipid accumulation in 3T3-L1 cells without showing cytotoxicity [21]. Comparable to the findings of Kowalska and coworkers, in our study, we observed a reduced lipid accumulation in 3T3-L1 cells for the fractions of chokeberry and cranberry extracts at concentrations of 25–50 µg/mL. Dallas et al. reported that a citrus-based polyphenolic dietary supplement (containing mainly cyanidin-3-glucoside and naringin) significantly stimulated the lipolytic activity of human adipocytes (ex vivo), and it showed a strong effect on cAMP-PDE inhibition [16]. We identified cyanidin-3-glucoside in the anthocyanin fractions of chokeberry, cranberry, and pomegranate extracts and observed inhibitory activities of these fractions towards PDE 3B. In addition, in our earlier study, it was demonstrated that peonidin-3-glucoside, cyanidin-3-arabinoside, and cyanidin-3-glucoside could inhibit PDE 3B activity with IC_50_ values between 120 ± 44 µM and 299 ± 56 µM [28]. In this study, we identified these anthocyanins in the anthocyanin fractions of chokeberry, cranberry, and pomegranate extracts and confirmed that the anthocyanin fractions are potent inhibitors of the enzyme PDE 3B.

Not only the anthocyanin fractions, but also the copigment fractions, showed a strong influence on the lipid metabolism. The anthocyanin fractions of the chokeberry and cranberry extracts contained both cyanidin-3-galactoside and cyanidin-3-arabinoside, while in the cranberry anthocyanin fraction, additionally, peonidin-3-galactoside was identified, whereas cyanidin-3,5-diglucoside and delphinidin-3-glucoside were the major compounds in the pomegranate anthocyanin fraction. It was shown that the fruit-dependent inhibition of PDE 3B activity and lipid accumulation is mainly due to the copigments, e.g., hydrolyzable tannins, which were found in the pomegranate copigment fraction and were efficient inhibitors of PDE 3B activity and lipid accumulation, whereas for the stimulation of lipolysis via FFA release, copigments like phenolic acids and flavonols were responsible.

Since the copigment fractions of cranberry and pomegranate extract showed strong effects on lipid metabolism in vitro, these two copigment fractions were further fractionated by HPCCC. Five subfractions (F1–F5) of the cranberry copigment fraction and seven subfractions (F1–F7) of the pomegranate copigment fraction were obtained. The first and second subfractions, F1 and F2, of the cranberry copigments contained mainly phenolic acids, such as caffeic acid, as well as hexosides of coumaric, ferulic, and sinapic acid. Subfraction F3 consisted of coumaric acid hexoside, myricetin hexoside, and coumaroyl iridoid hexoside, and subfraction F4 consisted of chlorogenic acid (hexoside), myricetin, and quercetin hexosides. The fifth subfraction (F5) included different quercetin derivates, coumaric acid, and myricetin-pentosides. It was more difficult to differentiate the subfractions of the pomegranate copigment extract. Punicalin occurred in the subfractions F1 and F2, punicalagin in the subfractions F1–F4, and pedunculagin in F1–F6. Ellagic acid hexoside was found in the subfractions F3–F5 and ellagic acid in F6 and F7. The last subfractions, F6 and F7, also included phenolic acids, such as caffeic acid, ferulic acid, and coumaric acid, and subfraction F7 included the flavonoids quercetin and luteolin hexoside.

All investigated subfractions prepared from the pomegranate copigment fraction by HPCCC reduced lipid accumulation in 3T3-L1 cells, whereas for the subfractions F2 and F3 of the cranberry copigment fraction, no influence on lipid accumulation was observed. The strongest inhibition of lipid accumulation could be detected for subfractions F3 and F4 of the pomegranate copigment fraction. Subfraction F3 consisted of ellagic acid hexoside, pedunculagin, and punicalagin, and subfraction F4 contained additionally granatin A and galloyl-O-punicalin. Similarly, Les et al. found that ellagic acid and punicalagin were able to reduce triglyceride accumulation in 3T3-L1 adipocytes [41]. Respectively, the strongest effects on lipid accumulation for the subfractions of the cranberry copigment fraction were shown by F1, F4, and F5. HPLC-ESI-MS/MS analysis of the HPCCC subfraction F1 revealed mainly caffeic acid, coumaroyl acid, and sinapic acid hexosides, whereas subfraction F4 contained chlorogenic acid and quercetin hexoside. Quercetin derivatives were enriched in subfraction F5, and the recorded activity could be linked to these compounds. In other studies, the influence of quercetin on the lipid metabolism in vitro was observed. Little et al. reported that a longer-term treatment for three sequential 24 h doses with low concentrations (*c* = 1 µM) of quercetin significantly lowered stored lipid content in human Simpson–Golabi–Behmel syndrome (SGBS) adipocytes [42]. In comparison to 3T3-L1 cells, SGBS expressed higher levels of adipocyte-specific transcripts during the process of differentiation, and differentiated SGBS expressed more comparable transcript levels, morphology, and biochemical functions to primary omental adipocytes [43].

The trends observed for the subfractions in the lipid accumulation assay were similar to those observed in the PDE activity assay. All investigated subfractions of the cranberry and pomegranate copigment fractions inhibited PDE 3B enzyme activity in vitro. Contrary to moderately active subfractions of the cranberry copigment fraction (IC_50_ = 37 *± 7* µg/mL–471 *±* 67 µg/mL), the subfractions of the pomegranate copigment fraction were more active, and the IC_50_ values ranged from 0.2 *±* 0.1 µg/mL to 9.0 *±* 0.1 µg/mL. Within the cranberry copigment subfractions under study, F5 was the most potent PDE 3B inhibitor. We identified quercetin derivatives in subfraction F5 and determined an IC_50_ value of 37 ± 7 µg/mL. Quercetin, which was tested for the effect on PDE from isolated rat adipocytes by Kuppusamy and Das, showed an inhibitory potency, with an EC_50_ value of 32 µM, and additionally a dose-dependent increase in lipolysis was observed [15]. The subfractions F2, F3, and F4 of the pomegranate copigment fraction demonstrated the strongest PDE 3B inhibition and contained primarily ellagic acid hexoside, pedunculagin, and punicalagin. Little has been published regarding the influence of these compounds on PDE inhibition. However, in our earlier study, pomegranate extracts, in which pedunculagin and punicalagin were identified as the major copigments, were already found to be potent inhibitors of the enzyme PDE 3B [28].

Our findings showed that the tested fractions and subfractions reduce lipid accumulation, increase lipolysis by FFA release, and inhibit PDE 3B activity in vitro, which are in vitro models and represent some parts of the human lipid metabolism. Therefore, the consumption of red fruit juices from chokeberry, cranberry, and pomegranate might influence lipid metabolism and help to prevent obesity. Since we did not investigate the effects of single compounds in the three in vitro assays (lipid accumulation, lipolysis, and PDE 3B activity), we cannot fully explain any finding and mechanism being responsible for the effects influencing lipid metabolism at present and must address this matter in future studies. Moreover, it could be helpful to reach a better separation of the copigments so that it could be possible in the future to determine their biological activity with single compounds.

Taken together, we were able to fractionate extracts (activity-guided) from fruit juice concentrates and could identify compounds in the fractions and subfractions, which are crucial for the effects on the lipid metabolism. However, the use of in vitro models provides only preliminary data, so further evaluation with additional studies, preferably in vivo, is essential, for example, by performing human intervention studies that investigate different parameters of lipid metabolism.

## Figures and Tables

**Table 1:** Polyphenol content in the fruit concentrates of chokeberry, cranberry, and pomegranate and also calculated for the juice dilution at reduced °Brix levels.

Concentrate	°Brix of Juice Concentrate	Polyphenolic Content of Juice Concentrates in g/100 mL	°Brix of Juice Dilution	Polyphenolic Content in Juice in g/100 mL
Chokeberry	65.4	4.08	18.0	1.12
Cranberry	49.8	1.39	8.8	0.25
Pomegranate	64.5	2.31	15.0	0.54

**Table 2:** Contents of anthocyanins and copigments in the extracts and total recovery.

Extract	Anthocyanin Content in g/100 g	Copigment Content in g/100 g	Recovery in g/100 g
Chokeberry	47.9	39.6	87.5
Cranberry	17.7	72.2	89.9
Pomegranate	14.4	79.8	94.2

**Table 3:** HPLC-ESI-MS/MS data, identified anthocyanins and their absorption maxima λ_max_, retention time of the compounds in the UHPLC-DAD chromatogram, and quantification with a calibration curve of cyanidin-3-glucoside as an equivalent of the chokeberry anthocyanin fraction. Compounds were identified by mass spectral data, comparisons with authentic references (marked with *), and literature data [30,31].

Peak No.	Retention Time (min)	[M-H]^+^ *m*/*z*	Fragments *m*/*z*	λ_max_	Anthocyanin	Concentration (mg/g)
1	6.2	737	575, 287	522	Cyanidin-3-hexoside-(epi)catechin	3.44 ± 0.09
2	7.4	707	575, 287	523	Cyanidin-3-pentoside-(epi)catechin	2.11 ± 0.05
3	9.1	449	287	515	Cyandin-3-galactoside *	328.26 ± 6.12
4	9.9	449	287	515	Cyanidin-3-glucoside *	18.06 ± 1.07
5	10.5	419	287	515	Cyanidin-3-arabinoside *	120.93 ± 4.71
6	12.4	419	287	517	Cyanidin-3-xyloside	23.20 ± 0.52
7	13.8	491	287	520	Cyanidin-derivative	17.69 ± 0.43

**Table 4:** HPLC-ESI-MS/MS data, identified copigments and their absorption maxima λ_max_, retention time of the compounds in UHPLC-DAD chromatogram, and concentrations calculated with a calibration curve as chlorogenic acid equivalents, for phenolic acids, or as quercetin-3-glucoside equivalents, for flavonols, in the chokeberry copigment fraction. Compounds were identified by mass spectral data, comparisons with authentic references (marked with *), and literature data [30,31].

Peak No.	Retention Time (min)	[M-H]^−^ *m*/*z*	Fragments *m*/*z*	λ_max_	Copigment	Concentration (mg/g)
1	3.6	153	109	295	Protocatechuic acid	0.29 ± 0.04
2	5.4	353	191,179,135	323	Neochlorogenic acid *	133.16 ± 4.35
3	7.1	461	337, 297, 275	310	Coumaroylquinic acid ester	3.49 ± 0.15
4	7.9	353	191, 179, 161	324	Chlorogenic acid *	118.90 ± 0.05
5	8.7	353	191	324	Cryptochlorogenic acid *	7.11 ± 0.41
6	9.7	367	335, 161, 132	323	Feruloylquinic acid	2.20 ± 0.08
7	10.2	595	385, 335, 235, 209	279	Unknown	7.27 ± 0.16
8	12.1	625	301	351	Quercetin-dihexoside	4.68 ± 0.25
9	12.7	625	301	349	Quercetin-dihexoside	10.01 ± 0.42
10	14.2	367	179, 135	-	Feruloylquinic acid	5.37 ± 0.27
11	14.7	595	301, 179, 151	352	Quercetin-3-vicianoside	15.39 ± 0.82
12	15.3	609	301	351	Quercetin-3-robinobioside	13.52 ± 0.79
13	15.5	609	301	349	Quercetin-3-rutinoside *	28.53 ± 1.03
14	15.8	463	301, 179	352	Quercetin-3-galactoside	13.95 ± 0.57
15	16.1	463	301, 151	352	Quercetin-3-glucoside *	18.83 ± 0.63
16	22.3	301	179, 151	-	Quercetin *	6.01 ± 0.18

**Table 5:** HPLC-ESI-MS/MS data, identified anthocyanins and their absorption maxima λ_max_, retention time of the compounds in the UHPLC-DAD chromatogram, and concentrations calculated with a calibration curve as cyanidin-3-glucoside equivalents of the cranberry anthocyanin fraction The compounds were identified by mass spectral data, comparisons with authentic references (marked with *), and literature data [32].

Peak No.	Retention Time (min)	[M+H]^+^ *m*/*z*	Fragments *m*/*z*	λ_max_	Anthocyanin	Concentration (mg/g)
1	9.2	449	287	515	Cyanidin-3-galactoside *	97.59 ± 5.11
2	9.9	449	287	524	Cyanidin-3-glucoside *	3.77 ± 0.23
3	10.5	419	287	516	Cyanidin-3-arabinoside *	92.97 ± 5.09
4	11.4	463	301	516	Peonidin-3-galactoside	141.96 ± 6.05
5	12.2	463	301	520	Peonidin-3-glucoside *	14.09 ± 0.87
6	12.7	433	301	516	Peonidin-3-arabinoside	70.72 ± 4.36
7	13.9	463	331	528	Malvidin-3-arabinoside	9.77 ± 0.48

**Table 6:** HPLC-ESI-MS/MS data, identified copigments and their absorption maxima λ_max_, retention time of the compounds in the UHPLC-DAD chromatogram, and concentrations calculated with a calibration curve as chlorogenic acid equivalents, for phenolic acids, or as quercetin-3-glucoside equivalents, for flavonols, of the cranberry copigment fraction. The compounds were identified by mass spectral data, comparisons with authentic references (marked with *), and literature data [32].

Peak No.	Retention time (min)	[M-H]^−^ *m*/*z*	Fragments *m*/*z*	λ_max_	Copigment	Concentration (mg/g)
1	6.0	341	179, 135	313	Caffeic acid hexoside	3.68 ± 0.12
2	7.4	325	163, 145, 117	315	Coumaric acid hexoside	6.75 ± 0.33
3	8.0	325	163, 145, 117	322	Coumaric acid hexoside	62.89 ± 3.91
4	8.8	353	191	322	Chlorogenic acid *	4.00 ± 0.15
5	9.1	355	193	328	Ferulic acid *	1.67 ± 0.09
6	9.7	577	407	308	Proanthocyanidin dimer	5.83 ± 0.34
7	10.3	385	223	294	Sinapic acid hexoside	3.41 ± 0.23
8	10.5	335	179	325	Caffeoylshikimic acid	4.94 ± 0.30
9	10.7	337	191	312	Coumaroylquinic acid	13.98 ± 0.68
10	12.6	863	711	310	Proanthocyanidin trimer	1.17 ± 0.09
11	13.2	479	316	354	Myricetin-hexoside	49.77 ± 1.87
12	13.9	449	316	354	Myricetin-xyloside	1.77 ± 0.10
13	14.1	493	330	357	Laricitrin-hexoside	2.27 ± 0.16
14	15.0	535	371	351	Coumaroyl Iridoid hexoside	8.95 ± 0.41
15	15.6	463	301	352	Quercetin-hexoside	104.73 ± 4.55
16	16.4	463	301	355	Quercetin-hexoside	6.71 ± 0.35
17	16.7	433	301	351	Quercetin-pentoside	13.78 ± 0.34
18	17.2	433	301	350	Quercetin-pentoside	60.66 ± 0.72
19	17.7	433	301	351	Quercetin-pentoside	21.13 ± 0.19
20	18.4	447	301	346	Quercetin-rhamnoside	25.35 ± 0.22
21	18.6	507	344	352	Syringetin-hexoside	24.06 ± 0.21
23	19.2	317	179	368	Myricetin *	8.32 ± 0.10
23	20.6	447	314	355	Isorhamnetin-pentoside	2.14 ± 0.10
24	21.6	477	344	351	Syringetin-pentoside	2.58 ± 0.11
25	22.3	301	179	368	Quercetin *	30.71 ± 0.87

**Table 7:** HPLC-ESI-MS/MS data, identified anthocyanins and their absorption maxima λ_max_, retention time of the compounds in the UHPLC-DAD chromatogram, and concentrations calculated with a calibration curve as cyanidin-3-glucoside equivalents of the pomegranate anthocyanin fraction. The compounds were identified by mass spectral data, comparisons with authentic references (marked with *), and literature data [33,34].

Peak No.	Retention Time (min)	[M+H]^+^ *m*/*z*	Fragments *m*/*z*	λ_max_	Anthocyanin	Concentration (mg/g)
1	6.3	627	303	523	Delphinidin-3,5-diglucoside	8.01 ± 0.29
2	7.5	611	287	514	Cyanidin-3,5-diglucoside *	22.51 ± 0.36
3	8.6	595	271	514	Delphinidin-3-glucoside *	7.36 ± 0.25
4	10.0	449	287	517	Cyanidin-3-glucoside *	16.89 ± 0.31

**Table 8:** HPLC-ESI-MS/MS data, identified copigments and their absorption maxima λ_max_, retention time of the compounds in the UHPLC-DAD chromatogram. And concentrations calculated with a calibration curve as punicalin equivalents, for hydrolyzable tannins, or as quercetin-3-glucoside equivalents, for flavonols, of the pomegranate copigment fraction. The compounds were identified by mass spectral data, comparisons with authentic references (marked with *), and literature data [33,34].

Peak No.	Retention Time (min)	[M-H]^−^ *m*/*z*	Fragments *m*/*z*	λ_max_	Copigment	Concentration (mg/g)
1	1.9	783	721, 601	377	Pedunculagin I	1.96 ± 0.11
2	2.1	1101	781, 601	377	Punicalin-derivative	9.77 ± 0.35
3	2.4	649	605, 301		Trisgalloyl-glucoside	7.15 ± 0.31
4	2.6	781	601, 271	378	Punicalin I *	40.36 ± 1.09
5	2.7	781	601, 299	377	Punicalin II *	53.44 ± 1.18
6	4.01	933	451	372	Galloyl-O-punicalin	37.17 ± 0.82
7	5.2	1083	601		Punicalagin I *	141.51 ± 3.60
8	6.9	951	907	373	Granatin B	12.25 ± 0.44
9	7.4	783	299, 601	376	Pedunculagin II	259.56 ± 10.79
10	8.0	469	425	371	Valonic acid bilactone	20.63 ± 0.58
11	8.3	951	783	377	HHDP-valoneoyl-glucoside	2.75 ± 0.14
12	8.6	799	301	376	Ellagic acid derivative	7.21 ± 0.26
13	8.9	1085	451	375	Digalloyl-galloyl-hexoside	1.96 ± 0.08
14	9.2	799	301	375	Granatin A	2.12 ± 0.09
15	9.3	325	145	312	Coumaric acid hexoside	6.31 ± 0.22
16	9.7	801	347	365	Digalloyl-HHDP-glucuronide	3.21 ± 0.20
17	9.8	449	287	322	Dihydrokaempferol-hexoside	7.81 ± 0.34
18	10.5	355	193	327	Ferulic acid hexoside	5.81 ± 0.29
19	10.7	633	301	370	Galloyl-HHDP-glucoside	9.59 ± 0.42
20	10.8	635	465	322	Tri-O-galloyl-glucoside	14.11 ± 0.57
21	12.1	463	301	360	Ellagic acid hexoside	29.09 ± 0.78
22	12.6	953	301	332	Galloyl-bis-HDDP-glucoside	12.78 ± 0.36
23	15.1	447	301	360	Quercetin-3-rhamnoside	66.68 ± 0.54
24	15.4	301	229	366	Ellagic acid *	13.53 ± 0.35

HHDP = hexahydroxydiphenic acid.

**Table 9:** The polymer content of the precipitates and of the extracts from chokeberry, cranberry, and pomegranate, calculated as cinnamtannin A2 equivalents with a calibration curve.

	Polymer Content (g/100 g)	
	Polymer Fraction after Precipitation	Extract
Chokeberry	37.85 ± 0.67	14.62 ± 0.05
Cranberry	41.23 ± 0.51	18.12 ± 1.03
Pomegranate	5.31 ± 0.12	2.60 ± 0.04

**Table 10:** Half-maximal inhibitory concentrations (IC_50_) of the three fractions (anthocyanin, copigment, and polymer) of the different extracts tested using the PDE 3B activity assay. Results are presented as the mean ± SD (*n* = 3–4).

Extract	Fraction	IC_50_ [µg/mL]
Chokeberry	anthocyanin	120 ± 6
	copigment	113 ± 15
	polymer	126 ± 13
Cranberry	anthocyanin	94 ± 13
	copigment	104 ± 10
	polymer	95 ± 20
Pomegranate	anthocyanin	13 ± 3
	copigment	12 ± 4
	polymer	10 ± 1

**Table 11:** The amount and yield of the subfractions F1–F5 and coil fraction (F6) obtained after HPCCC separation of the cranberry copigment fraction.

Subfraction	F1	F2	F3	F4	F5	F6 (Coil)	Sum
Amount (g)	0.72	0.48	0.26	0.24	0.20	0.11	2.01
Yield (%)	32.7	21.8	11.8	10.9	9.1	5.0	91.3

**Table 12:** The amount and yield of subfractions obtained after HPCCC separation of the pomegranate copigment fraction.

Fraction	F1	F2	F3	F4	F5	F6	F7 (Coil)	Sum
Amount (g)	1.08	0.58	1.03	0.90	0.34	0.83	0.66	5.42
Yield (%)	17.6	9.4	16.7	14.7	5.5	13.5	10.7	88.1

**Table 13:** Identified compounds in the HPCCC subfractions of the cranberry and pomegranate copigment fractionations, according to the results in Section 3.2.

Subfraction	Cranberry	Pomegranate
F1	Caffeic acid hexoside, Caffeic acid derivative, Coumaric acid hexoside, Sinapic acid hexoside	Punicalin, Pedunculagin, Punicalagin, Punicalagin like
F2	Caffeic acid hexoside, Coumaric acid hexoside, Ferulic acid hexoside, Sinapic acid hexoside, Coumaroyl Iridoid hexoside	Punicalin, Pedunculagin, Galloyl-O-punicalin, Punicalagin, Punicalagin derivative
F3	Coumaric acid hexoside, Myricetin hexosid, Coumaroyl Iridoid hexoside	Ellagic acid hexoside, Pedunculagin, Punicalagin
F4	Chlorogenic acid, Chlorogenic acid hexoside, Quercetin hexoside, Laricitrin hexoside	Ellagic acid hexoside, Pedunculagin, Galloyl-O-punicalin, Granatin A, Punicalagin
F5	Coumaric acid, Myricetin-pentosides, Proanthocyanidin dimer, Quercetin-hexosides, Quercetin	Ellagic acid hexoside, Pedunculagin, Punigluconin, HHDP-valoneoyl hexoside, Digalloyl-gallagyl hexosid
F6		Ellagic acid, Ferulic acid hexoside, HHDP hexoside, Galloyl-HHDP hexoside, Tri-O-galloyl hexoside, Pedunculagin, Digalloyl-HHDP hexoside, Granatin A, Granatin B, Galloyl-bis-HDDP hexoside
F7 (Coil)		Caffeic acid, Ellagic acid, Coumaric acid hexoside, Quercetin, Luteolin hexoside, Valonic acid dilactone, Digalloyl-HHDP hexoside, Tetra-O-galloyl hexoside, Granatin A, HHDP-valoneoyl hexoside, Granatin B

With HHDP = hexahydroxydiphenic acid.

**Table 14:** Half-maximal inhibitory concentrations (IC_50_) of the subfractions (F1–F5) of the cranberry copigment fraction. Results are presented as means ± SD (*n* = 3–4).

Fraction	Subfraction	IC_50_ [µg/mL]
Cranberry copigment fraction	F1	45 ± 15
	F2	266 ± 10
	F3	471 ± 67
	F4	229 ± 23
	F5	37 ± 7

**Table 15:** Half-maximal inhibitory concentrations (IC_50_) of the subfractions (F1–F7) of the pomegranate copigment fraction. Results are presented as means ± SD (*n* = 3–5).

Fraction	Subfraction	IC_50_ [µg/mL]
Pomegranate copigment fraction	F1	4.8 ± 1.6
	F2	0.5 ± 0
	F3	0.2 ± 0.1
	F4	0.5 ± 0.2
	F5	3.8 ± 1.2
	F6	8.5 ± 3.0
	F7	9.0 ± 0.1

## Data Availability

Data is contained within the article.
